# Editorial: Unlocking the potential of extracellular vesicles in tumor immunity and drug resistance

**DOI:** 10.3389/fonc.2026.2031751

**Published:** 2026-10-27

**Authors:** Yu Fu, Tianzhao Du, Huizhe Xu, Shenglong Li

**Affiliations:** 1 Department of Neurosurgery, Cancer Hospital of Dalian University of Technology, Liaoning Cancer Hospital and Institute, Shenyang, Liaoning, China; 2 Central Laboratory, Cancer Hospital of Dalian University of Technology, Liaoning Cancer Hospital and Institute, Shenyang, Liaoning, China; 3 Second Ward of Bone and Soft Tissue Tumor Surgery, Cancer Hospital of Dalian University of Technology, Liaoning Cancer Hospital and Institute, Shenyang, Liaoning, China; 4 School of Biomedical Sciences, Faculty of Medicine, The Chinese University of Hong Kong(CUHK), Hong Kong, Hong Kong SAR, China

**Keywords:** drug resistance, extracellular vesicle, immunity resistance, tumor microenvironment, tumor treatment

Extracellular vesicles (EVs) are increasingly recognized as important mediators of intercellular communication in tumors. Current research is no longer limited to describing their molecular cargo but has gradually shifted toward understanding the roles of EVs in tumor state evolution, immune responses, and the regulation of therapeutic responses, as well as their potential use in therapeutic interventions. The studies included in this Research Topic mainly focus on three interrelated themes: the relationships between EVs and tumor progression, cellular plasticity, and heterogeneity; the roles of EVs in tumor immune regulation and therapeutic resistance; and the therapeutic applications of treatment-induced and engineered EVs. Collectively, these studies indicate that EVs play a dual role: they serve as important carriers mediating adaptive changes in tumors while also holding potential as tools for antitumor therapy.

The first theme focuses on the relationships among EV biology, tumor state evolution, and cellular heterogeneity. Xu et al. reviewed EV-mediated intercellular crosstalk in hepatocellular carcinoma (HCC), highlighting how noncoding RNAs, proteins, and other bioactive molecules can be transported via vesicles to influence tumor growth, metabolism, invasion, metastasis, and interactions with the stroma. Li et al. identified an ETV5-driven C3 ID4+ melanoma cell subpopulation characterized by more pronounced stemness-related features, enhanced oxidative phosphorylation, reduced immune-related programs, and active TGF-β signaling. Fan et al. identified an ASS1+ malignant plasma cell population enriched in relapsed/refractory multiple myeloma, characterized by high metabolic activity, EV-related transcriptional features, and extensive intercellular communication with myeloid cells and dendritic cell populations. These two single-cell studies primarily inferred the involvement of EVs based on transcriptomic features and intercellular communication patterns. Therefore, further direct analyses of vesicle production, cargo composition, and their effects on recipient cells are still needed. At a broader mechanistic level, Liu et al. described the bidirectional regulatory relationship between EV biology and acetylation modifications, providing a theoretical framework for understanding the links between vesicle-mediated intercellular communication and metabolic, transcriptional, and epigenetic plasticity. Overall, these studies suggest that EVs not only participate in the transport of specific molecular cargo but may also play an important role in linking tumor heterogeneity with adaptive changes in cellular states.

The second theme focuses on the roles of EVs in regulating antitumor immunity and therapeutic resistance. Wang et al. reviewed how glioma-derived EVs can reprogram multiple immune cell populations through checkpoint molecules, noncoding RNAs, metabolites, and other regulatory cargo, thereby promoting T-cell dysfunction, immunosuppressive myeloid phenotypes, impaired antigen presentation, and resistance to immunotherapy. In HCC, Xu et al. similarly highlighted EV-associated mechanisms that influence macrophage polarization, lymphocyte function, cell survival signaling, and resistance to systemic therapies, including sorafenib and lenvatinib. Extending this concept to another disease context, Yang et al. proposed an ecosystem model of chemoresistance in osteosarcoma, in which chemotherapy-surviving tumor cells, EV-mediated communication, and microenvironmental remodeling interact across treatment and metastatic niches. Collectively, these studies suggest that therapeutic resistance is a multicellular adaptive process in which EVs can coordinate tumor-intrinsic survival programs with immune and stromal remodeling.

The same biological properties that enable EVs to transport tumor-promoting signals can also be exploited for therapeutic purposes. Ortiz-Bonilla et al. demonstrated that the internalization of Bacillus Calmette-Guérin (BCG) by bladder cancer cells increased EV release and altered EV cargo, resulting in EVs enriched in antigen-presentation and costimulatory molecules. These EVs activated dendritic cells, enhanced T-cell responses, and improved BCG efficacy in a syngeneic model. These findings highlight the potential role of treatment-induced EVs as active mediators of antitumor immunity in the specific context of BCG therapy. From an engineering perspective, Fang et al. reviewed exo-nanomaterials that combine the biocompatibility and trafficking properties of EV membranes with the loading capacity and programmability of synthetic nanomaterials. Such platforms may enable the delivery of immune agonists, checkpoint-modulating agents, neoantigens, and nucleic acid vaccines, although most evidence remains preclinical. Xu et al. similarly discussed the use of engineered EVs to deliver regulatory RNAs to restore therapeutic sensitivity and promote treatment-associated cell death. Collectively, these approaches represent a conceptual shift from inhibiting pathological EV signaling to deliberately redirecting EV-mediated communication for therapeutic purposes.

In summary, this Research Topic highlights the context-dependent roles of EVs in cancer. Future studies should move beyond the descriptive profiling of EV cargo to further elucidate the causal relationships among specific tumor cell states, EV subpopulations, recipient cells, and treatment conditions. In particular, EV-associated transcriptomic signatures should be distinguished from experimentally demonstrated vesicle-mediated functions. Furthermore, the cellular origin, biodistribution, and recipient cell specificity of functional EV populations require more precise characterization. For clinical translation, standardized EV characterization, scalable production, reproducible cargo loading, and rigorous evaluation of safety and *in vivo* behavior will also be essential. Addressing these challenges will help clarify when EV-mediated signaling should be inhibited, monitored, or utilized for therapeutic purposes, ultimately supporting the development of more precise EV-based strategies for cancer treatment.

